# Bone Organoids as Advanced Models for Osteoporosis: Development, Application, and Future Prospects

**DOI:** 10.3390/ijms27073118

**Published:** 2026-03-30

**Authors:** Chao Liu, Xueliang Zhang, Rui Yu

**Affiliations:** The First Clinical College of Shenyang Campus, Liaoning University of Traditional Chinese Medicine, Shenyang 110847, China; liuc199702@163.com (C.L.); zhangxl210001@163.com (X.Z.)

**Keywords:** bone organoids, osteoporosis, bone remodeling, translational medicine, stem cell technology

## Abstract

The prevalence of osteoporosis, a skeletal disorder characterized by reduced bone mass, microarchitectural deterioration, and increased fracture risk, poses a substantial global healthcare burden. Although animal models and two-dimensional cell cultures have been used to advance bone research, they do not completely replicate the multicellular interactions, extracellular matrix organization, and biomechanical environment of human bone, limiting their translational relevance. This review provides a critical synthesis of recent advances in bone organoid technology, emphasizing biological complexity, technical innovation, and relevance to osteoporosis modeling. Beyond summarizing progress, we distinguish validated capabilities from aspirational claims and identify the methodological gaps that must be addressed before bone organoids can reliably support drug screening, regenerative medicine, and precision approaches. Advances in stem cell biology, tissue engineering, and three-dimensional culture systems have enabled the use of self-organizing, multicellular organoids that reproduce key physiological and pathological features of bone. These systems model estrogen-deficiency-induced bone loss, glucocorticoid-associated osteoporosis, aging-related degeneration, and genetic susceptibility. By integrating osteogenic and endothelial components within biomimetic matrices, bone organoids can support mechanistic studies and pharmacological testing. However, their incomplete vascularization, limited mechanical fidelity, instability, and lack of standardized benchmarks restrict their translational readiness. Overcoming these barriers requires technological refinement, quantitative metrics, and regulatory alignment.

## 1. Introduction

Osteoporosis is a prevalent and debilitating systemic skeletal disorder characterized by reduced bone mass and deterioration of bone microarchitecture, leading to compromised bone strength and an elevated risk of fractures. This condition disproportionately affects the elderly and imposes a substantial global healthcare burden. Epidemiological estimates indicate that osteoporosis impacts over 200 million people worldwide [1], with osteoporotic fractures severely reducing quality of life, increasing mortality, and generating enormous economic costs due to prolonged hospitalization, rehabilitation, and ongoing medical care.

Traditionally, insights into osteoporosis pathogenesis and treatment development have relied heavily on animal models and two-dimensional (2D) cell culture systems. Rodent models, particularly genetically modified mice, have elucidated many molecular pathways underlying bone metabolism and remodeling [2]. However, these models possess critical limitations, including interspecies differences, ethical concerns, high maintenance costs, and limited translational relevance due to their inability to fully replicate human bone physiology and pathology. Similarly, 2D cultures of osteoblasts, osteoclasts, or progenitor cells, while convenient and high-throughput, lack the three-dimensional (3D) architecture, extracellular matrix composition, and cell–cell interactions inherent to native bone, constraining their predictive power in preclinical research [3].

Recent advances in stem cell biology and tissue engineering have enabled the development of bone organoids—sophisticated, miniaturized 3D structures derived from stem or progenitor cells that replicate key aspects of bone development, organization, and function in vitro—that hold great promise for overcoming these traditional limitations [4]. These constructs incorporate diverse bone-relevant cell types—including osteoblasts, osteoclasts, osteocytes, endothelial cells, and mesenchymal stem cells—within an extracellular matrix, thus reproducing the cellular heterogeneity and structural complexity of native bone tissue.

Some figures in this manuscript were generated with the assistance of artificial intelligence (AI) tools. The authors have carefully reviewed, validated, and approved all content of these figures, and take full responsibility for their accuracy, integrity, and compliance with academic standards.

A notable milestone in this field was the development of a trabecular bone organoid prototype which combined human osteoblasts and osteoclasts on bovine-derived micro-trabeculae, cultured within microgravity-based bioreactors to simulate a dynamic, mechanically relevant microenvironment [5]. More recently, micron-scale trabecular bone organoids incorporating human osteoblastic and osteoclastic cells have been developed to better model the human bone remodeling microenvironment while avoiding the limitations of highly mineralized in vivo tissue and the ethical constraints of animal models [5,6].

Moreover, the emergence of human pluripotent stem cells (hPSCs), including human embryonic stem cells (hESCs) and induced pluripotent stem cells (hiPSCs), has created unprecedented opportunities for generating patient-specific bone organoids [7]. These can be used to precisely model various genetic and acquired forms of osteoporosis, promoting personalized approaches to disease research and therapy.

Innovations in bioengineering, such as scaffold-based and scaffold-free systems, have further improved the development of bone organoids [8]. Scaffold-based strategies employ biocompatible materials—including hydrogels, polymeric scaffolds, and ceramic composites—to support 3D tissue formation, while scaffold-free approaches harness self-assembly or microgravity culture to enable intrinsic cell organization. Advanced technologies such as bioprinting additionally enable precise spatial patterning and vascularization, closely replicating the complex anatomical and physiological features of native bone [9].

Overall, bone organoids are a transformative platform for studying the pathophysiology of osteoporosis, in addition to drug discovery and regenerative medicine in the field [10]. By accurately replicating the 3D microenvironment and multicellular interactions of bone tissue, these models hold tremendous promise for improving translational research and personalized therapeutic strategies for osteoporosis.

## 2. Development of Bone Organoids: Strategies and Key Milestones

Over the past decade, fueled by progress in stem cell biology, bioengineering, and biomaterials science, bone organoid research has significantly advanced [10]. Early strategies in this field primarily utilized mesenchymal stem cells (MSCs), typically derived from bone marrow or adipose tissue, seeded within biocompatible scaffolds composed of collagen, hydroxyapatite, or poly(lactic-co-glycolic acid) (PLGA) [11]. These scaffolds created a supportive three-dimensional environment promoting cell attachment, proliferation, and osteogenic differentiation, especially when combined with osteoinductive factors such as BMP-2 or dexamethasone [12], laying the groundwork for more sophisticated tissue-engineered bone models [13].

The application of human pluripotent stem cells (hPSCs), including human embryonic stem cells (hESCs) and induced pluripotent stem cells (hiPSCs), was a major breakthrough that enabled the creation of patient-specific and genetically customized bone organoids [14]. Stepwise differentiation protocols modulating the BMP/TGF-β, Wnt, Hedgehog, and Notch signaling pathways have enhanced the reproducibility and disease relevance of organoids, opening avenues for personalized disease modeling [4]. Further innovations include the use of structurally and biochemically relevant substrates, such as demineralized bone paper (DBP), which preserves collagen alignment and mechanical durability [4]. These platforms support osteoblast mineralization, bone-lining cell phenotypes, and transitions between active and resting states. Modular ring-based systems have also enabled spatiotemporal studies of local remodeling processes [5].

Scaffold-free strategies have also advanced the field by enabling cells to self-organize through intrinsic cell–cell adhesion and extracellular matrix deposition [15]. Devices and methods such as hanging-drop cultures, microwell arrays, and microgravity bioreactors have facilitated the development of spheroidal or tubular bone-like tissues that better reflect developmental bone morphogenesis and preserve endogenous signaling cues [16].

An additional transformative milestone is bioprinting [12]. By depositing bioinks composed of living cells and hydrogels in a layer-by-layer fashion, osteogenic, endothelial, and even neural cells can be constructed in precise spatial patterns [17], promoting prevascularization and enhancing nutrient diffusion. This technology also supports the incorporation of spatial gradients of growth factors and mineral components to more accurately replicate the heterogeneity of native bone [9]. Recent studies have devised centimeter-scale, patient-customized constructs with high cell viability and rapid osteointegration [18]. Further developments include combining hPSCs with dynamic culture systems and fine-tuned biomaterials to better reflect hierarchical organization and spatiotemporal remodeling [19]. For example, the integration of human MSCs, human umbilical vein endothelial cells (HUVECs), and osteogenic microparticles within bioprinted constructs produced prevascularized, centimeter-scale bone organoids with rapid osteointegration [20].

Synthetic biology approaches have added yet another dimension to organoid development. Bacterial extracellular vesicles engineered to deliver osteoinductive and angiogenic factors, co-encapsulated with immunomodulatory cytokines, have been shown to orchestrate complex signaling within bone organoids [21].

Innovative hydrogel systems, such as dual-network hydrogels combining DNA and GelMA with biomimetic mitochondrial nanominerals and catalase, possess tunable viscoelasticity and biochemical cues that support woven bone organoid formation via intramembranous ossification [22]. Similarly, chondrogenic priming followed by endochondral ossification has produced vascularized, mineralized constructs that model estrogen-deficiency osteoporosis [23].

Highly biomimetic scaffolds, such as decellularized–decalcified matrices with reconstructed vascular channels, have been used to recreate the compartment-specific architecture of cortical and cancellous bone [24].

Other advanced strategies have leveraged hydroxyapatite nanoparticles incorporated into MSC spheroids to promote osteogenic differentiation and self-organization into trabecular-like constructs [25]. Advanced 3D bioprinting combined with projection-based light curing has been used to generate precisely controlled microstructures that support osteogenic differentiation, mineralization, and vascularization after in vivo implantation [26].

**Figure 1:**
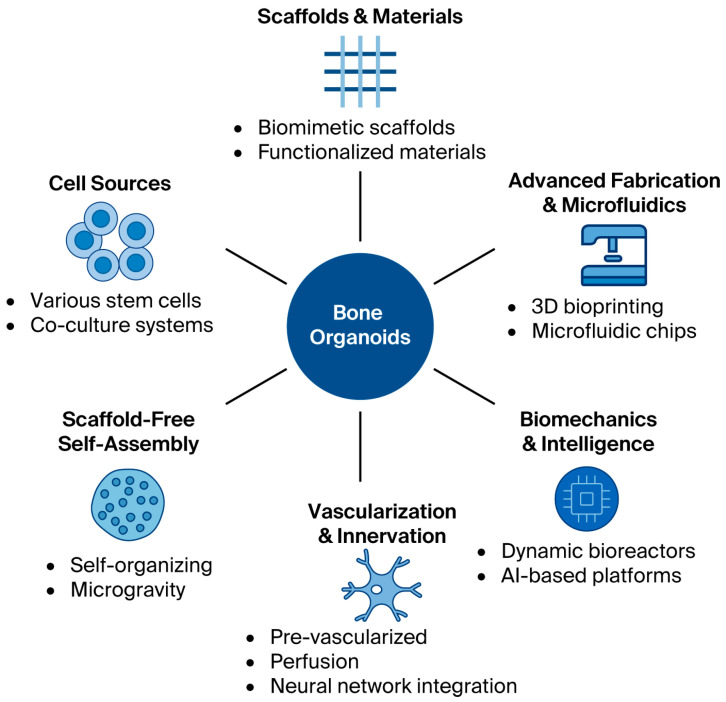
Strategies and technological innovations for engineering bone organoids. This schematic summarizes key strategies and advances supporting the development of bone organoids as physiologically relevant models. Six major domains are highlighted: (1) diverse cell sources, including mesenchymal stem cells (MSCs), human pluripotent stem cells (hPSCs), and iPSC-derived multicellular co-cultures; (2) biomimetic scaffolds and functionalized materials, incorporating collagen, hydroxyapatite, PLGA, decellularized–decalcified matrices, tunable hydrogels, and mineral nanoparticles to mimic native bone architecture; (3) advanced fabrication and microfluidics, leveraging 3D bioprinting, micro-scale patterning, gradient deposition of bioactive factors, and microfluidic perfusion systems; (4) scaffold-free self-assembly techniques such as hanging-drop cultures, microwell arrays, and microgravity bioreactors to enable spontaneous tissue morphogenesis; (5) vascularization and innervation strategies using endothelial co-cultures, prevascularized scaffolds, perfusion systems, and neural network integration to better replicate native bone physiology; and (6) biomechanical and intelligent platforms that combine dynamic bioreactor mechanical loading with artificial intelligence, high-throughput screening, and automated biomanufacturing for scalable, standardized production. Collectively, these integrated approaches support the development of reliable and translational bone organoid models for osteoporosis research and regenerative therapies.

Mechanical loading is also increasingly recognized as vital for developing functional bone organoids. Dynamic bioreactors applying cyclic compression within 3D bioprinted scaffolds have enhanced osteocyte differentiation, lacunar–canalicular network formation, and collagen fiber maturation while improving the mechanical strength of the constructs [27]. Nevertheless, vascularization remains a persistent challenge. Early milestones included co-culturing endothelial progenitor cells to improve perfusion [28], while newer strategies have explored microfluidic chips and controlled angiogenic factor release to promote vascular network formation [29]. We illustrate the strategies and technological innovations in the field of engineering bone organoids in Figure 1.

The field is also moving toward standardization, automation, and reproducibility. Integrating artificial intelligence to optimize differentiation protocols and adopting high-throughput screening platforms for drug testing will be critical for clinical translation [30]. Collectively, these milestones establish a robust foundation for the future of bone organoids as reliable, physiologically relevant disease models and regenerative medicine platforms.

## 3. Cellular and Molecular Complexity of Bone Organoids

The cellular and molecular complexity of bone organoids underpins their potential as realistic in vitro models of the physiology and pathology of human bone [31]. Bone organoids typically incorporate multiple cell types, including osteoblasts, osteoclasts, osteocytes, endothelial cells, stromal cells, and progenitor cells [32]. This cellular heterogeneity enables the recreation of intricate physiological interactions critical for bone homeostasis, remodeling, and responses to pathological conditions such as osteoporosis [33].

Osteoblasts, derived from mesenchymal stem cells (MSCs), play a central role in synthesizing bone matrix proteins—including collagen type I, osteocalcin, and osteopontin—and are regulated by signaling pathways such as Wnt/β-catenin, BMP, and TGF-β [34]. In parallel, osteoclasts, originating from hematopoietic precursors, drive bone resorption through secretory and proteolytic mechanisms, with their differentiation predominantly regulated by the RANKL–RANK–OPG axis [35]. This coupling is effectively modeled in advanced bone organoid platforms [36]. Osteocytes, embedded within the mineralized bone matrix, function as mechanosensory cells coordinating remodeling in response to mechanical and hormonal signals [37]. Their inclusion in bone organoids supports the exploration of mechanotransduction pathways, contributing to our understanding of bone density regulation and osteoporosis development [38].

Endothelial cells incorporated into bone organoids mimic vascular networks essential for nutrient delivery, oxygen transport, and waste removal [39]. This vascularization improves the physiological relevance of organoids and enables the study of angiogenesis, its crosstalk with osteogenesis, and the vascular deficiencies observed in osteoporosis [40].

Recent advances in single-cell RNA sequencing and spatial transcriptomics have enabled the unprecedented mapping of cellular heterogeneity in bone organoids [41]. These tools have been used to reveal the transcriptional signatures of subpopulations and cell–cell signaling dynamics, enhancing our understanding of how bone tissue maintains homeostasis and deteriorates in disease states [42,43].

The major molecular pathways extensively characterized in bone organoids include Wnt/β-catenin signaling [44], critical for osteoblast differentiation and function; RANKL–RANK–OPG signaling, essential for osteoclastogenesis [44]; BMP/TGF-β signaling, which supports osteoblast and chondrocyte lineage commitment [45]; and Notch signaling, implicated in maintaining skeletal homeostasis [46].

Beyond classical pathways, advanced bone organoid systems have demonstrated dynamic paracrine and juxtacrine regulatory networks. For example, Park et al. highlighted vitamin D3- and prostaglandin E2-driven modulation of RANKL and OPG secretion, along with Connexin 43-mediated gap junction signaling [5]. Deng et al. designed microsphere-based bone organoids incorporating oxygen-releasing particles and macrophages, promoting M2 polarization and dynamically regulating TGF-β, Wnt, PI3K/Akt, and focal adhesion pathways [47].

Other studies have shown that bone organoids express critical regulatory proteins such as sclerostin (SOST), parathyroid hormone receptor 1 (PTHR1), and Connexin 43, highlighting their ability to replicate the sophisticated signaling processes involved in bone remodeling [6,31]. Zhu et al. demonstrated that woven bone organoids activated MAPK pathways and promoted osteogenic gene expression in a reactive oxygen species-scavenging environment [22], while Duan et al. found that graphene oxide-laden microparticles activated the PI3K/Akt and focal adhesion pathways, enhancing extracellular matrix structure and osteogenic gene programs [48].

Further, metabolic signals have been shown to influence bone organoids. Jiang et al. demonstrated that 3-hydroxybutyrate activates HCAR2 receptors, elevates intracellular calcium, and triggers a cAMP/PKA/CREB cascade, promoting osteogenic transcription factor expression and anti-apoptotic proteins [49].

**Figure 2:**
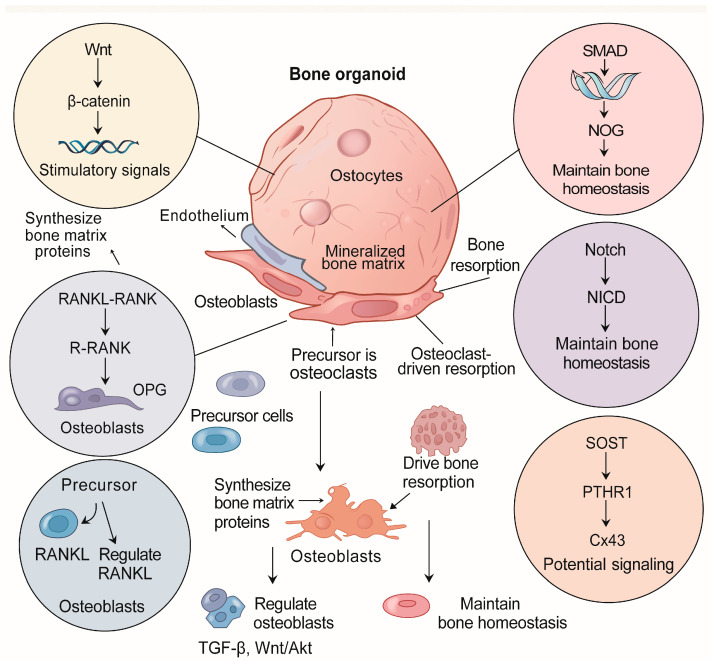
Cellular heterogeneity and key molecular signaling pathways in bone organoids. This schematic illustrates the multicellular composition of bone organoids, including osteoblasts, osteoclasts, osteocytes, endothelial cells, stromal cells, and macrophages, and depicts their interconnected signaling pathways. The major pathways represented include Wnt/β-catenin, BMP/TGF-β, RANKL–RANK–OPG, Notch, MAPK, and PI3K/Akt, which regulate osteogenesis, osteoclastogenesis, and bone remodeling. Additional paracrine signals such as vitamin D3, prostaglandin E2, Connexin 43-mediated gap junctions, and metabolic cues (e.g., 3-hydroxybutyrate) are highlighted for their roles in coordinating bone cell crosstalk and maintaining bone homeostasis. The illustration also emphasizes the contributions of mechanical loading and the vascular–immune microenvironment to bone organoid function and disease modeling.

Fuller et al. developed a multicellular 3D-mcBOM platform that captured osteoblast–osteoclast coupling with Runx2, Sp7, Col1a1, and TRAP expression, supporting matrix deposition and osteocyte lacunae formation [50]. Toni et al. created a vasculomorphic bone organoid reproducing compartment-specific differences in mineralization and osteogenic gene activation between cortical and cancellous bone [51]. In Figure 2, we demonstrate the cellular heterogeneity and key molecular signaling pathways present in bone organoids.

Collectively, these findings underscore the robust capacity of bone organoids to recapitulate complex, physiologically relevant multicellular and molecular networks [52]. Such models provide a valuable basis for investigating osteoporosis pathogenesis, therapeutic screening, and regenerative applications [10].

## 4. Use of Bone Organoids as Models to Study Pathogenesis of Osteoporosis

Bone organoids have rapidly emerged as powerful tools for investigating osteoporosis, a multifactorial skeletal disorder characterized by reduced bone density and compromised microarchitecture [1]. Traditional in vivo and two-dimensional in vitro models fail to reproduce the complex, dynamic cell–cell and cell–matrix interactions within human bone tissue [53]. In contrast, bone organoids can reproduce key aspects of the bone microenvironment, providing an advanced platform to explore the pathogenesis of osteoporosis [31].

One of the most widely studied mechanisms driving osteoporosis is estrogen deficiency [54]. Postmenopausal osteoporosis, primarily linked to estrogen withdrawal, leads to increased osteoclastogenesis, enhanced bone resorption, and reduced osteoblast activity [55]. Bone organoids have also enabled in-depth analyses of estrogen signaling, revealing changes in RANKL/OPG ratios, Wnt pathway disruptions, and altered cytokine profiles [56].

Similarly, glucocorticoid-induced osteoporosis (GIO), a prevalent form of secondary osteoporosis, is caused by chronic glucocorticoid administration, which suppresses osteoblast proliferation and promotes apoptosis [57]. Bone organoids provide a controlled 3D microenvironment to study glucocorticoid receptor signaling; previous studies have highlighted disruptions in the BMP/TGF-β and Wnt/β-catenin pathways and identified potential therapeutic targets [58].

Age-related osteoporosis involves a progressive loss of bone density with advancing age, driven by cellular senescence, chronic inflammation, and reduced regenerative capacity of bone progenitors [59]. Bone organoids derived from aged donor cells or engineered to mimic aging phenotypes have helped to clarify mechanisms such as the senescence-associated secretory phenotype (SASP), inflammation-induced resorption, and decreased osteogenic responsiveness [60].

Genetic risk factors also play a role in osteoporosis susceptibility [60]. The combination of CRISPR–Cas9 genome editing with bone organoid systems allows the polymorphisms implicated in bone density regulation to be precisely modeled, including variants in WNT16, LRP5, and TNFRSF11B [61,62], thereby advancing personalized medicine strategies [63].

Beyond modeling pathogenesis, woven bone organoids based on CGDE hydrogels have shown functional in vivo osteointegration and mineralization, suggesting potential for simulating impaired mineralization and testing osteogenic therapies [64]. Fuller et al. reported that their 3D-mcBOM platform could simulate osteoporosis-like conditions by adjusting culture media and applying metabolic or inflammatory stressors, reflecting imbalances between resorption and formation [50].

Patient-derived iPSC-based bone organoids have revolutionized individualized osteoporosis models [65]. They enable patient-specific disease mechanisms and differential drug responses to be evaluated, supporting precision therapeutic development [24]. Similarly, a trabecular bone organoid platform developed using demineralized bone paper supports high-content imaging and multiplex molecular analysis, facilitating detailed observation of osteoblast–osteoclast interactions and remodeling dynamics [5]. Duan et al. showed that prevascularized bone organoids integrating graphene oxide, MSCs, and HUVECs enhanced bone formation and vascularization even in large-scale defects, indicating their potential for studying impaired osteogenesis and microvascular dysfunction related to osteoporosis [48]. Deng et al. demonstrated that their microsphere-based platform, combining immunomodulatory macrophages with oxygen-release systems, could be used to model bone healing deficits associated with osteoporosis [47].

In a vascularized bone organoid system, Bukhari et al. (2025) reported dynamic remodeling under estrogen-deficient conditions, including sclerostin upregulation, altered apoptosis pathways, and CD31^+^ vessel-like structures, mimicking osteoporosis-related mineralization heterogeneity [23]. Toni et al. developed a flat and short bone-mimicking organoid which reproduced compartment-specific differences between cortical and cancellous sites, applicable to site-specific osteoporotic lesions [51]. Li et al. (2024) developed a hydroxyapatite–MSC microtissue model that reflected impaired mineralization and unbalanced osteoblast–osteocyte differentiation when FAK/Akt signaling was suppressed, providing a valuable in vitro model for osteoporosis-related remodeling disturbances [25].

**Figure 3:**
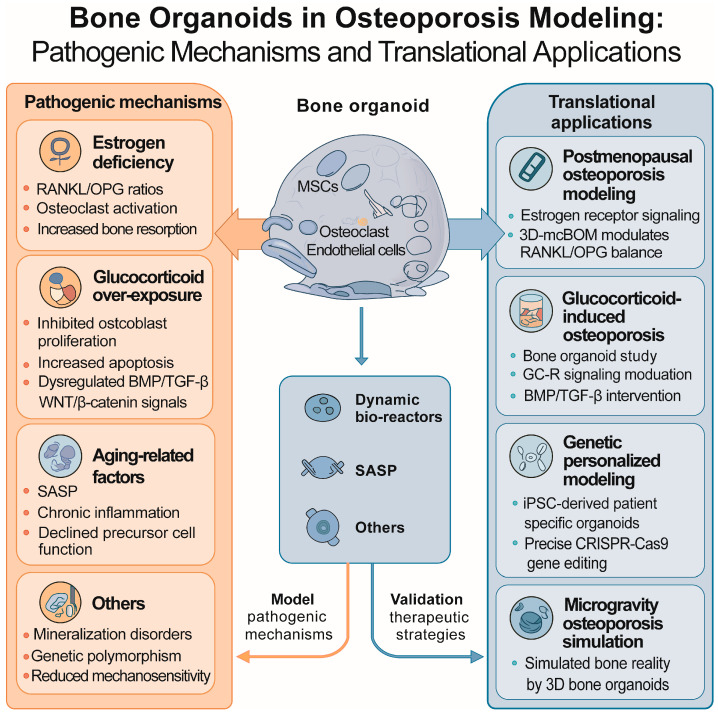
Modeling osteoporosis pathogenesis and therapeutic applications using bone organoids. This schematic summarizes how bone organoids can serve as advanced in vitro models to investigate multiple pathophysiological mechanisms of osteoporosis, including estrogen deficiency, glucocorticoid overexposure, aging-related degeneration, and genetic susceptibility. It also highlights their potential to simulate mineralization disorders, vascular dysfunction, and microgravity-induced bone fragility. The right-hand side illustrates how these organoids enable personalized therapeutic testing, regenerative engineering, and advanced drug screening, thus representing a translational bridge between basic research and clinical innovation.

Finally, trabecular bone organoids cultivated under microgravity conditions have been used to reproduce pathological bone loss patterns, including altered osteoclast resorption and reduced mechanical resistance, reflecting the fragility seen in osteoporosis. Such approaches may help translate basic discoveries into clinically meaningful strategies. Figure 3 summarizes how bone organoids are applied to model the pathogenesis of osteoporosis and evaluate therapeutic strategies.

In summary, bone organoids offer unprecedented opportunities to model the pathogenesis of osteoporosis, explore disease-specific mechanisms, and test targeted interventions. These platforms are likely to play an increasingly important role in precision medicine approaches to osteoporosis management.

## 5. Applications in Therapeutic Screening and Regenerative Research

Bone organoids are a versatile and powerful platform for therapeutic screening and regenerative medicine, showing particular promise for advancing the treatment of osteoporosis [66]. Their sophisticated architecture and high physiological relevance offer distinct advantages over conventional in vivo and in vitro models, enabling more precise assessments of drug efficacy, toxicity, and mechanisms of action specific to osteoporotic contexts [67].

In therapeutic osteoporosis screening, bone organoids have greatly accelerated drug discovery pipelines [68]. High-throughput screening (HTS) platforms integrated with organoid systems can facilitate the rapid identification and validation of pharmacological candidates while closely reproducing human osteoporotic bone responses [69]. These models have been successfully employed to test anabolic agents such as teriparatide, romosozumab, and abaloparatide, as well as anti-resorptive drugs including bisphosphonates and denosumab [70,71]. Furthermore, bone organoids provide a valuable framework for studying monoclonal antibodies, growth factor therapies, and gene-editing strategies targeting bone metabolism pathways relevant to osteoporosis [72]. Their multicellular complexity and incorporation of vascular-like structures enable these interventions to be evaluated in a physiologically relevant and osteoporotic-like environment [73]. As such, bone organoids serve as a critical translational bridge between preclinical osteoporosis research and clinical applications [74].

In regenerative medicine, bone organoids hold significant promise for developing innovative approaches to repairing and reconstructing osteoporotic bone [75]. Their structural resemblance to native bone positions them as potential candidates for tissue-engineered grafts, personalized implants, and autologous transplantation, particularly for patients with osteoporosis [76]. Patient-derived iPSC-based bone organoids reduce immunogenicity risks while enabling precision regenerative therapies tailored to osteoporotic patients [77]; additionally, they offer unique opportunities to examine the regeneration mechanisms relevant to osteoporosis, including stem cell niche alterations, osteogenic differentiation pathways, and angiogenic responses in compromised bone [78].

Recent studies have integrated bone organoids into more complex tissue-engineered constructs to enhance regenerative outcomes specifically for osteoporotic conditions [79]. For example, Duan et al. developed prevascularized, 3D bioprinted bone organoids that supported the evaluation of osteogenic and angiogenic therapeutics, advancing patient-specific regenerative treatments for critical-size osteoporotic bone defects [48]. Deng et al. showed that microsphere-based conformal bone organoids combining immunomodulatory macrophages with oxygen-release systems enhanced angiogenesis and osteogenesis, highlighting their potential in osteoporotic bone regeneration [47]. Furthermore, Bukhari et al. (2025) developed a 3D vascularized bone organoid platform for testing anti-resorptive and pro-angiogenic osteoporosis therapies, recapitulating estrogen-deficiency-induced alterations in mineralization and vascularization under human-relevant conditions [23]. The trabecular bone organoid model developed by Iordachescu et al. demonstrated compatibility with high-throughput assays and microCT quantification, supporting its use in drug screening and fracture healing studies [6].

The translational potential of bone organoids for osteoporosis in therapeutic screening and regenerative applications is substantial [68]. Advancements in automation, robotics, and artificial intelligence are expected to improve the scalability, reproducibility, and predictive capabilities of these models. Close collaboration between bioengineers, stem cell scientists, clinicians, and industry stakeholders will be essential for achieving these goals. Ultimately, bone organoids are poised to transform osteoporosis management through precision medicine and personalized therapeutic strategies.

## 6. Current Limitations and Technical Challenges

Despite this remarkable progress, the applications of bone organoids in osteoporosis research and therapeutic development still face significant technical and conceptual challenges [80]. One primary limitation is the incomplete replication of the bone microenvironment, particularly in terms of vascularization and innervation [81]: functional blood vessels are essential for delivering nutrients, oxygen, and regulatory signals, while innervation contributes to mechanosensory feedback and remodeling processes [82]. Current bone organoids often lack fully mature and functional vascular and neural networks, reducing their physiological fidelity and limiting long-term viability [83].

Another limitation is the insufficient representation of mechanical loading. In vivo, bone tissue is continuously subjected to complex mechanical forces that influence cell differentiation, matrix deposition, and overall bone strength [84,85]. However, the existing bone organoid systems rarely replicate these dynamic biomechanical stimuli. To address this limitation, advanced bioreactors capable of applying physiologically relevant compression, tension, and shear forces must be developed [86].

Long-term culture stability and reproducibility further limit the translational potential of bone organoids. Variability in stem cell sources, differentiation protocols, and culture conditions often leads to inconsistent maturation, hindering scalability and standardization [74,87]. Moreover, the lack of robust quality control frameworks makes cross-laboratory reproducibility difficult, creating obstacles to clinical translation [69]. Ethical and regulatory considerations present additional challenges, especially regarding the use of patient-derived iPSCs [88]. Issues related to informed consent, data privacy, and ownership of biological materials require well-defined ethical and legal guidelines to support safe and responsible clinical use [89].

Another recurrent issue is the scalability of high-throughput drug screening and large-scale regenerative applications [90]. Current bone organoid fabrication processes are labor- and resource-intensive, which limits their practicality in industrial or widespread clinical use [91]. Advances in robotics, automation, and artificial intelligence-assisted biomanufacturing will be essential to address these issues.

Specific limitations in existing trabecular bone organoid models have also been noted. For instance, Iordachescu et al. highlighted the need to further refine the osteoblast-to-osteoclast ratio and investigate mechanotransduction pathways such as Connexin 43 signaling under unloading conditions [24]. Park et al. (2021) noted a lack of osteocytes and dynamic mechanical loading within their DBP-based organoids, which constrained their ability to mimic physiological strain [5]. Duan et al. emphasized the challenges of controlling graphene oxide degradation, ensuring consistent vascular integration, and balancing immune responses in future clinical translation [48].

Collectively, these gaps highlight key areas for continued innovation to fully recapitulate the complexity of in vivo bone remodeling. Addressing these challenges will require close interdisciplinary collaboration between bioengineers, stem cell scientists, clinicians, ethicists, and regulatory agencies. Ultimately, overcoming these barriers is critical to realizing the transformative promise of bone organoids in osteoporosis research, precision therapy, and regenerative medicine [70,71].

## 7. Current Capabilities Versus Aspirational Goals: Benchmarking the Translational Readiness of Bone Organoids

### 7.1. Experimentally Validated Capabilities of Current Bone Organoid Platforms

Despite substantial conceptual and technological progress, only a defined subset of bone organoid functions has been experimentally validated using reproducible and quantitative endpoints [6,73]. First, osteoblast–osteoclast coupling represents one of the most consistently supported capabilities. Multiple platforms, including trabecular bone organoids, demineralized bone paper-based systems, and multicellular three-dimensional bone organoid models, have demonstrated reproducible reciprocal regulation between bone formation and resorption [5,6,50]. Quantifiable readouts, such as alkaline phosphatase activity, collagen type I deposition, mineralized matrix formation, and TRAP-positive osteoclast differentiation, support the presence of coordinated remodeling behavior rather than isolated lineage-specific responses [6]. Second, matrix mineralization and structural maturation have been reliably achieved across both scaffold-based and bioprinted systems [22,48]. Objective measurements, including calcium deposition, collagen organization, and microCT-derived mineral density, provide quantitative evidence that these constructs can recapitulate selected structural features of bone tissue formation [48]. Third, directionally consistent responses to disease-relevant perturbations have been demonstrated. Estrogen withdrawal, glucocorticoid exposure, inflammatory stress, and aging-associated cues induce predictable shifts in RANKL/OPG balance, osteoblast survival, and osteoclast activity, qualitatively aligning with the established mechanisms of osteoporosis pathogenesis [23,50]. Collectively, these capabilities define the currently validated experimental core of bone organoid technology.

### 7.2. Aspirational but Not Yet Fully Validated Capabilities

Functional vascularization is commonly inferred from endothelial marker expression or the presence of CD31^+^ structures. However, key hallmarks of physiological vasculature, including sustained perfusion, flow-dependent remodeling, and long-term vessel stability, have not yet been systematically demonstrated in most platforms [40,48]. Similarly, innervation and neuro–bone coupling, while conceptually compelling, lack standardized integration strategies and quantitative functional readouts [61,92]. Physiologically relevant mechanical loading also remains incompletely realized. Although cyclic compression or shear stress have been applied in selected bioreactor systems, the multiaxial strain patterns and adaptive mechanotransduction responses characteristic of native bone tissue have not yet been robustly reproduced or standardized across platforms [70,93]. Addressing this gap will require integration of advanced bioreactor engineering with quantitative strain mapping and standardized mechanical benchmarks.

### 7.3. Quantitative Benchmarking: Comparison with Existing Models

Our direct comparison of established experimental systems highlights both the strengths and current limitations of bone organoids [31,54,60], as presented in Table 1 below, which contains a comparative benchmarking of bone organoids, two-dimensional cell cultures, and animal models used in osteoporosis research.

At present, bone organoids do not outperform animal models in predictive accuracy for clinical outcomes such as fracture risk. Their principal advantage lies in enhanced human relevance and mechanistic resolution, positioning them as complementary rather than replacement models within the current collection of preclinical research.

### 7.4. Failed or Incomplete Solutions: Lessons from Current Platforms

Several widely explored strategies illustrate persistent methodological and translational limitations. For example, uncontrolled self-assembly in scaffold-free systems frequently leads to pronounced batch-to-batch heterogeneity, thereby compromising reproducibility and quantitative comparability [94]. Over-mineralized constructs, while structurally robust, impair nutrient diffusion, imaging resolution, and dynamic remodeling capacity [95]. Endothelial co-culture approaches lacking perfusion typically generate transient, non-functional vascular structures [48,96]. In parallel, mechanical loading systems often lack standardized strain calibration, limiting cross-study comparability and quantitative interpretation [97].

These examples underscore a critical insight: increased technical complexity does not inherently confer functional fidelity.

### 7.5. Pathways Toward Translational and Regulatory Readiness

For bone organoids to support clinical translation and drug development, future efforts must prioritize standardized manufacturing workflows, quantitative quality control metrics, and automation-compatible platforms [98]. It is equally important to validate them against clinically meaningful endpoints, including bone mineral density surrogates and fracture-risk-associated biomarkers [87]. Alignment with the regulatory frameworks governing advanced medicinal products and therapies will be essential to ensure safety, reproducibility, and scalability, particularly as bone organoid systems move toward translational and industrial contexts [99].

Taking the above into account, bone organoids represent a promising but still evolving platform for osteoporosis research. While they provide superior human relevance and mechanistic insight compared with conventional models, their current applications are best suited for mechanistic validation and medium-throughput screening rather than industrial-scale drug discovery. Addressing unresolved challenges, particularly vascularization, mechanical fidelity, reproducibility, and quantitative benchmarking, will be critical to realizing their full translational potential.

## 8. Discussion

Looking forward, bone organoids hold tremendous promise for advancing osteoporosis research, diagnostics, and therapeutic strategies. Future efforts should prioritize overcoming current technical limitations through the integration of emerging technologies and interdisciplinary collaboration. A critical direction is the enhancement of vascularization and innervation within bone organoids [100]. Methods to reliably establish mature, functional vascular and neural networks will improve their physiological relevance and translational potential [101,102]. Emerging bioengineering advances such as microfluidic devices, 3D bioprinting, and co-culture systems offer promising routes through which to achieve these goals.

Additionally, the ability to simulate mechanical loading will be a crucial area of future developments. Advanced bioreactor systems capable of applying physiological compressive, tensile, and shear forces will improve bone organoids’ ability to model bone remodeling and osteoporosis-associated pathology [86,103], while integrating high-throughput screening platforms and automation will further enhance their scalability, reproducibility, and standardization [69]. Artificial intelligence and computational modeling also hold transformative potential, enabling the optimization of differentiation protocols, prediction of patient-specific disease trajectories, and improvement of therapeutic screening accuracy [104,105].

**Figure 4:**
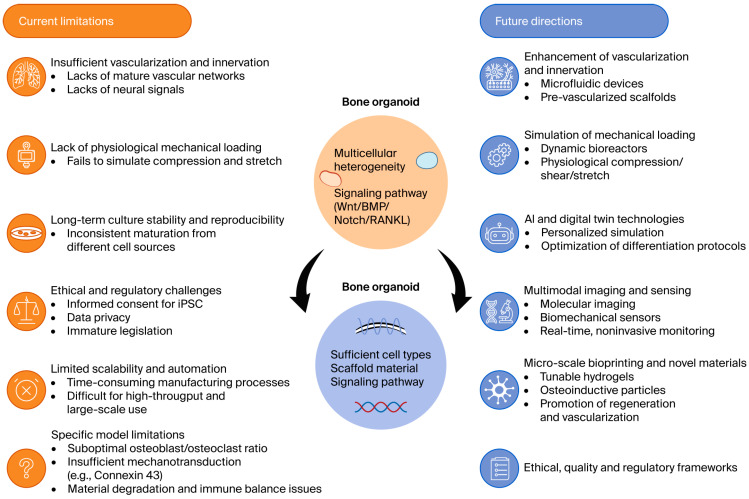
Challenges and future perspectives of bone organoids in osteoporosis research. This schematic illustrates the current limitations of bone organoid models, including incomplete vascularization and innervation, lack of mechanical loading, long-term stability, ethical and regulatory concerns, and scalability barriers. It also summarizes future research directions, highlighting the integration of advanced bioreactors, microfluidic perfusion, artificial intelligence, digital twin technologies, and standardization frameworks. These approaches are expected to enhance the translational relevance of bone organoids, advancing osteoporosis diagnostics, therapeutics, and regenerative medicine.

Combining organoid data with digital twin technologies—virtual representations of a patient’s bone physiology that update with real-time clinical and molecular data—could enable extremely precise simulations of disease progression and treatment responses [106]. Furthermore, multimodal imaging technologies such as high-resolution microscopy, molecular imaging, and biomechanical sensors will enable comprehensive, noninvasive, and longitudinal evaluations of bone organoid structure and function [107]. Finally, close cooperation between researchers, clinicians, engineers, regulatory authorities, and industry partners will be essential. Establishing robust ethical guidelines, standardized quality control systems, and appropriate regulatory frameworks will help accelerate the safe and effective clinical translation of bone organoid technologies [108]. In Figure 4, we conclude by addressing the major challenges and future perspectives of bone organoids in the context of osteoporosis research.

In conclusion, bone organoids offer a valuable and increasingly sophisticated experimental system for studying osteoporosis-related biology. Their greatest current strength lies in bridging mechanistic insight and human relevance. Indeed, bone organoids are positioned as superior alternatives to traditional 2D cultures for osteoporosis research due to their ability to model multicellular interactions, structural complexity, and disease-specific pathophysiology. To realize their translational potential, future development must converge on a standardized validation framework. This benchmark should be multi-dimensional, assessing structural, functional, and molecular fidelity, with predictive validity—the accurate forecasting of human clinical drug responses—as the ultimate criterion.

Continued progress will depend on rigorous benchmarking, transparent reporting of limitations, and coordinated efforts toward standardization and regulatory alignment.

## Figures and Tables

**Table 1:** Comparative benchmarking of bone organoids, two-dimensional cell cultures, and animal models in osteoporosis research.

Dimension	Bone Organoids	2D Cell Culture	Animal Models
Predictive validity (drug response direction)	Moderate (qualitative concordance)	Low	High
Throughput	Low–moderate	High	Low
Inter-laboratory reproducibility	Variable	High	Moderate
Cost per experimental condition	Moderate–high	Low	High
Human biological relevance	High	Low	High

## Data Availability

No new data were created or analyzed in this study. Data sharing is not applicable to this article.

## Abbreviations

The following abbreviations are used in this manuscript:

2D	two-dimensional
hPSCs	human pluripotent stem cells
hESCs	human embryonic stem cells
hiPSCs	induced pluripotent stem cells
MSCs	mesenchymal stem cells
PLGA	poly(lactic-co-glycolic acid)
DBP	demineralized bone paper
HUVECs	human umbilical vein endothelial cells
SOST	sclerostin
PTHR1	parathyroid hormone receptor 1
SASP	senescence-associated secretory phenotype
